# Outbreak of SARS-CoV-2 B.1.1.7 Lineage after Vaccination in Long-Term Care Facility, Germany, February–March 2021

**DOI:** 10.3201/eid2708.210887

**Published:** 2021-08

**Authors:** Pinkus Tober-Lau, Tatjana Schwarz, David Hillus, Jana Spieckermann, Elisa T. Helbig, Lena J. Lippert, Charlotte Thibeault, Willi Koch, Leon Bergfeld, Daniela Niemeyer, Barbara Mühlemann, Claudia Conrad, Stefanie Kasper, Friederike Münn, Frank Kunitz, Terry C. Jones, Norbert Suttorp, Christian Drosten, Leif Erik Sander, Florian Kurth, Victor M. Corman

**Affiliations:** Charité–Universitätsmedizin Berlin, Berlin, Germany (P. Tober-Lau, T. Schwarz, D. Hillus, E.T. Helbig, L.J. Lippert, C. Thibeault, W. Koch, L. Bergfeld, D. Niemeyer, B. Mühlemann, C. Conrad, S. Kasper, F. Münn, T.C. Jones, N. Suttorp, C. Drosten, L.E. Sander, F. Kurth, V.M. Corman);; Paritätisches Seniorenwohnen gGmbH, Berlin (J. Spieckermann);; German Centre for Infection Research (DZIF), Berlin (D. Niemeyer, B. Mühlemann, T.C. Jones, C. Drosten, V.M. Corman);; Bezirksamt Lichtenberg von Berlin, Berlin (F. Kunitz);; University of Cambridge, Cambridge, UK (T.C. Jones);; German Center for Lung Research, Gießen, Germany (N. Suttorp, L.E. Sander);; Bernhard Nocht Institute for Tropical Medicine (F. Kurth);; University Medical Centre Hamburg-Eppendorf, Hamburg, Germany (F. Kurth)

**Keywords:** vaccine, mRNA, outbreak, phylogeny, immunity, T cell, B cell, antibody, COVID-19, coronavirus disease, SARS-CoV-2, severe acute respiratory syndrome coronavirus 2, viruses, respiratory infections, zoonoses, Berlin, Germany

## Abstract

One week after second vaccinations were administered, an outbreak of B.1.1.7 lineage severe acute respiratory syndrome coronavirus 2 infections occurred in a long-term care facility in Berlin, Germany, affecting 16/20 vaccinated and 4/4 unvaccinated residents. Despite considerable viral loads, vaccinated residents experienced mild symptoms and faster time to negative test results.

Outbreaks of severe acute respiratory syndrome coronavirus 2 (SARS-CoV-2) in long-term care facilities (LTCF) are of great concern and have been reported to have high case-fatality rates (*1*). Consequently, national vaccination strategies prioritize residents of LTCFs (*2*).

The coronavirus disease (COVID-19) mRNA vaccine BNT162b2 (Pfizer-BioNTech, https://www.pfizer.com) has demonstrated high efficacy against COVID-19 (*3*). Protection has been observed >12 days after the first vaccination, and reported vaccine efficacy is 52% between the first and second dose and 91% in the first week after the second dose (*3*). Although breakthrough infections have been reported, vaccinated persons were at substantially lower risk for infection and symptomatic disease (*4*,*5*).

The variant of concern (VOC) B.1.1.7 rapidly became the predominant lineage in Europe in 2021. Analyses estimated that B.1.1.7 has increased transmissibility and a <0.7 higher reproduction number (*6*). Neutralization activity of serum samples from BNT162b2-vaccinated persons has been shown to be slightly reduced against B.1.1.7 in cell culture (*7*), but observational data from Israel suggest BNT162b2 vaccination is effective against B.1.1.7 (*8*).

We investigated a SARS-CoV-2 B.1.1.7 outbreak in a LTCF, which involved 20 BNT162b2-vaccinated residents and 4 unvaccinated residents. We report on clinical outcomes, viral kinetics, and control measures applied for outbreak containment. The study was approved by the ethics committee of Charité–Universitätsmedizin Berlin (EA2/066/20) and conducted in accordance with the Declaration of Helsinki and guidelines of Good Clinical Practice (https://www.ema.europa.eu/en/documents/scientific-guideline/ich-e-6-r2-guideline-good-clinical-practice-step-5_en.pdf).

## The Study

**Figure 1:**
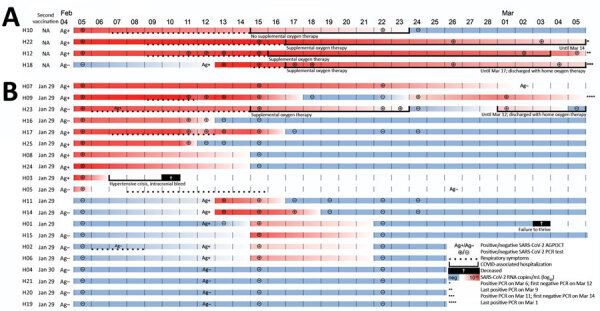
Individual trajectories of 24 long-term care facility residents over 30-day study period in outbreak of SARS-CoV-2 B.1.1.7 lineage infections, Germany, February–March 2021. A) Four unvaccinated residents; B) 20 residents who received their second dose of BNT162b2 COVID-19 mRNA vaccine (https://www.pfizer.com) on January 29 or 30, 2021. After a positive result in a healthcare worker, residents received AgPOCT and subsequently underwent regular RT-PCR testing for SARS-CoV-2. Dotted lines indicate respiratory symptoms, and continuous lines indicate hospitalization. AgPOCT, antigen point-of-care test; COVID-19, coronavirus disease; RT-PCR, reverse transcription PCR; SARS-CoV-2, severe acute respiratory syndrome coronavirus 2.

On February 4, 2021, daily SARS-CoV-2 screening of employees yielded a positive antigen point-of-care test (AgPOCT) result in 1 caregiver in a LTCF in Berlin, Germany. Among 24 residents of the unit under their responsibility, 20 (83%) residents had received the second dose of BNT162b2 on January 29 or 30, 2021 (Figure 1). Four residents had not been vaccinated for nonmedical reasons (i.e., personal refusal or delayed provision of consent by legal guardian). AgPOCTs and reverse transcription PCR (RT-PCR) testing of all residents on February 4 detected SARS-CoV-2 infections in 3/4 unvaccinated and 10/20 vaccinated residents (Figure 1). At the time of testing, 2 vaccinated patients exhibited mild fatigue and one of those also had diarrhea; all other patients were asymptomatic.

The next week, testing detected 7 additional infections, resulting in 4/4 unvaccinated infected residents and 16/20 vaccinated infected residents. The remaining 4 vaccinated residents tested negative throughout the 30-day observation period (Figure 1).

In addition to residents, 11/33 (33%) staff members from the unit tested positive for SARS-Cov-2 by February 18; of those, none were twice-vaccinated staff members, 2/8 (25%) had received 1 dose of BNT162b, and 9/22 (40.9%) had not been vaccinated. No infected staff required hospital treatment.

**Figure 2:**
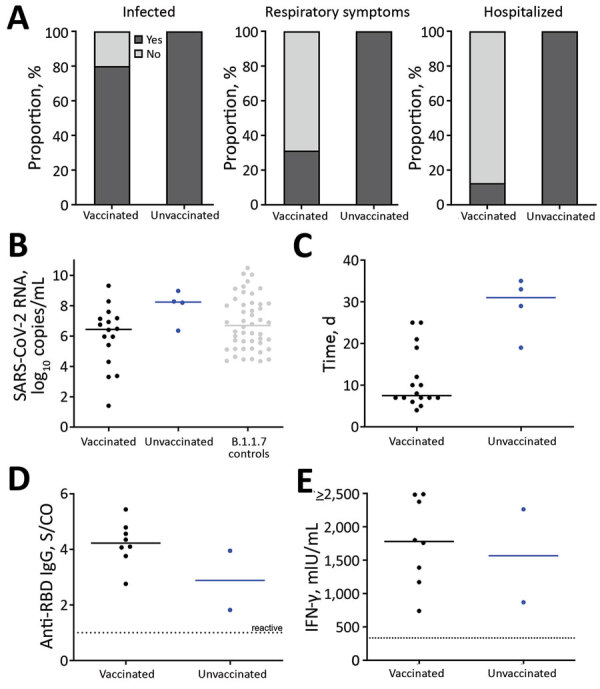
Characteristics of outbreak of SARS-CoV-2 B.1.1.7 lineage infections after vaccination in long-term care facility, Germany, February–March 2021. A) After a positive test result in a healthcare worker, 16/20 (80.0%) vaccinated residents and 4/4 (100.0%) unvaccinated residents subsequently tested positive for SARS-CoV-2. Among infected patients, 5/16 (31.25%) vaccinated and all 4 (100.0%) unvaccinated patients exhibited respiratory symptoms (i.e., cough or shortness of breath) during the course of disease. All 4 unvaccinated patients required hospital treatment; 3 (75.0%) received supplemental oxygen therapy and a standard course of dexamethasone. Two (12.5%) vaccinated patients also required hospital treatment, including 1 patient who experienced hypertensive crisis and intracranial bleeding and died 4 days after admission, and 1 patient with secondary bacterial pneumonia and urinary tract infection. B) Peak SARS-CoV-2 RNA concentrations in infected vaccinated residents (n = 16) and infected unvaccinated residents (n = 4), as well as SARS-CoV-2 B.1.1.7 RNA concentrations of an independent group of age-matched persons (n = 48) without known vaccination status whose infections were diagnosed during routine care. C) Time between first positive and first negative reverse transcription PCR or antigen point-of-care test result in vaccinated (n = 16) and unvaccinated (n = 4) residents. In 3 residents (2 vaccinated and 1 unvaccinated), negativity was determined by antigen point-of-care test only. D) Anti-SARS-CoV-2 receptor binding domain–specific IgG. E) IFN-γ release assay of SARS-CoV-2 specific T cells measured in 10/20 (50.00%) vaccinated and 2/4 (50.00%) unvaccinated residents 5 weeks after initial testing. IFN-γ, interferon-γ; SARS-CoV-2, severe acute respiratory syndrome coronavirus 2; S/CO, signal-to-cutoff ratio.

Respiratory symptoms, including cough and shortness of breath, occurred in 5/16 (31.3%) vaccinated patients and all 4 unvaccinated patients (Figure 2, panel A; Appendix Table). All 4 unvaccinated SARS-CoV-2–infected patients and 2/16 (12.5%) vaccinated patients required hospitalization (Figure 1; Figure 2, panel A). Supplemental oxygen therapy was required by 3/4 (75.0%) unvaccinated and 1/16 (6.3%) vaccinated patients (Figure 1; Figure 2, panel A). Two patients, 1/16 (6.3%) vaccinated persons and 1/4 (25.0%) unvaccinated persons, required intermittent oxygen therapy after discharge. One vaccinated patient with a history of hypertension and microvascular dementia died 6 days after testing positive by RT-PCR because of a hypertensive crisis with intracerebral hemorrhage. Another vaccinated patient died 16 days after testing positive by RT-PCR. Neither patient experienced respiratory symptoms during the infection (Figure 1).

Containment measures in place included mandatory use of FFP2 or N95 masks and daily AgPOCT screening for anyone entering the facility. Immediately after detection, the facility was closed to visitors and additional containment measures were put in place, including designated staff and separate entrance, elevator, and changing rooms. Staff were required to change personal protective equipment before entering each room. Residents of all 7 units of the LTCF underwent weekly AgPOCT for >3 weeks, and residents in the adjacent unit underwent AgPOCT every 2–3 days. The outbreak was contained within the unit; no further cases were detected.

All SARS-CoV-2 RNA-positive samples were tested for presence of SARS-CoV-2 VOCs by RT-PCR and complete genome sequencing (Appendix). RT-PCR suggested the presence of B.1.1.7, which was confirmed by sequencing in 11 patients for whom sufficient sequence information was available. In phylogenetic analysis, sequences form a monophyletic clade with additional sequences from Berlin interspersed (Appendix Figure 1), suggesting a common outbreak source, including infections outside the unit.

We performed serial RT-PCR testing of nasopharyngeal swab specimens from 22 patients. SARS-CoV-2 RNA concentrations peaked within 5 days (Appendix Figure 2). The median peak SARS-CoV-2 RNA concentration in vaccinated and unvaccinated patients overlapped concentrations detected at time of diagnosis in B.1.1.7 patients of similar ages (Figure 2, panel B). However, SARS-CoV-2 RNA concentration was lower among vaccinated residents than unvaccinated residents, although the difference was not statistically significant (6.45 vs. 8.15 log_10_ copies/mL; p = 0.10). Furthermore, duration of SARS-CoV-2 RNA shedding was considerably shorter in vaccinated patients than in unvaccinated patients (7.5 [95% CI 7–17.3] days vs. 31 [95% CI 21.5–34.5] days; p = 0.003) (Figure 2, panel C). Peak SARS-CoV-2 RNA concentrations above 10^6^ copies per mL, below which virus isolation in cell culture is usually not successful, were detected in all 4 unvaccinated patients but only in 7/16 vaccinated patients (*9*).

We further assessed the level of infectiousness in 22 samples from 14 patients by virus cell culture (Appendix). One sample obtained from a vaccinated patient 7 days after the first positive RT-PCR test, which showed 9.32 log_10_ SARS-CoV-2 RNA copies/mL, yielded a positive isolation outcome. Isolation attempts from samples of the same patient taken in the next 4 days and from 21 samples taken from 13 other patients were unsuccessful.

Five weeks after initial testing, 8/8 vaccinated and infected residents and 2/2 unvaccinated and infected residents showed robust antibody responses against SARS-CoV-2 spike antigens, virus neutralization capacity, and interferon-γ release of SARS-CoV-2–specific T cells (Figure 2, panels D, E; Appendix Figure 3). These results confirm the immune response capability in these patients.

## Conclusions

We performed a longitudinal study of SARS-CoV-2 infections in a LTCF unit. Nearly all infected residents were symptomatic, including most residents that had received a second BNT162b2 dose the week before. The outbreak was caused by SARS-CoV-2 VOC lineage B.1.1.7, which might partly explain the high attack rate and lack of protection in vaccinated residents. Nevertheless, we reported a lower attack rate, a shorter duration of SARS-CoV-2 RNA shedding, and a lower proportion of symptomatic COVID-19 requiring hospitalization and oxygen support for vaccinated patients. However, despite the limited sample size and the short interval between second vaccination and infection, this outbreak raises questions about the effectiveness of the vaccination regimen in the elderly (*3*,*8*,*10*–*12*). A delayed and overall reduced immune response to BNT162b2 vaccination has been described in elderly persons (*13*,*14*), which might explain the reported outbreak and infections in LTCF described elsewhere (*4*,*5*).

This outbreak highlights that older adults have reduced protection <2 weeks after second BNT162b2 vaccination. Therefore, single-dose regimens and extended dosing intervals might be insufficient for fully protecting this population (*15*). Vaccination of LTCF residents and staff is likely effective in reducing the spread of SARS-CoV-2. However, regular SARS-CoV-2 screening, prompt outbreak containment, and nonpharmaceutical interventions (*16*) remain necessary for optimal protection in this setting.

Appendix Additional information about outbreak of SARS-CoV-2 B.1.1.7 lineage after vaccination in long-term care facility, Germany, February–March 2021
