# Supplementary material for: Outbreak of SARS-CoV-2 B.1.1.7 Lineage after Vaccination in Long-Term Care Facility, Germany, February–March 2021
Source: Emerg Infect Dis. 2021 Aug;27(8):2169–73. doi: 10.3201/eid2708.210887 (PMC8314829; doi:10.3201/eid2708.210887)
Supplement: Appendix — Additional information about outbreak of SARS-CoV-2 B.1.1.7 lineage after vaccination in long-term care facility, Germany, February–March 2021 [file 21-0887-Techapp-s1.pdf]

Article DOI: https://doi.org/10.3201/eid2708.210887

Outbreak of SARS-CoV-2 B.1.1.7 Lineage after Vaccination in Long-Term Care Facility, Germany, February–March 2021

Appendix

Patients, Materials and Methods

Subjects were enrolled in the Pa-COVID-19 study conducted at Charité - Universitätsmedizin Berlin, a prospective observational study on the pathophysiology of coronavirus disease (COVID-19) (*1*). The Pa-COVID-19 study is registered in the German and the World Health Organization international registry for clinical studies (DRKS00021688). Written informed consent was obtained from all patients or legal representatives. Additional investigation of nasopharyngeal and oropharyngeal swabs was performed in accordance with §25 of the Berlin State Hospital Law, allowing for pseudonymized analysis of routine patient data by the treating physicians.

SARS-CoV-2 Screening

We performed rapid antigen point-of-care tests (AgPOCT) (NADAL COVID-19 Ag Schnelltest [Nal von minden, https://www.nal-vonminden.com] and MEDsan SARS-CoV-2 Antigen Rapid Test [MaiMed, https://maimed.de]) (*2*), and patients affected by the outbreak received regular oropharyngeal or nasopharyngeal swabs (daily, every 2–3 days, or weekly, depending on the patients’ willingness) throughout the study period. Swab specimens were immediately stored in 2 mL of viral transport medium at −20°C.

Real-Time Reverse Transcription PCR for SARS-CoV-2 and Typing PCR

RNA was extracted by using the MagNApur 96 DNA and viral NA small volume Kit (Roche, https://www.roche.com) on a MagNA Pure 96 System as recommended by the manufacturer. Real time reverse transcription PCR (rRT-PCR) was performed targeting the envelope (E) gene and nucleocapsid (N) gene on the Roche Light Cycler 480 system (Tib-Molbiol, https://www.tib-molbiol.de). Viral loads in throat swab specimens were given as

SARS-CoV-2 copies/mL diluted swab specimen. rRT-PCR used targets in the E and N genes on the Roche Light Cycler 480 system (Tib-Molbiol). Viral loads in throat swab specimens were presented as SARS-CoV-2 copies/mL diluted swab. Assessment of SARS-CoV-2 RNA concentration was done by applying external or internal calibration curves and quantified SARS-CoV-2 RNA and by using serial diluted specific in vitro–transcribed RNA standards as previously described (*3*–*5*). A probe-based melting curve assay (Tib-Molbiol) for SARS-CoV-2 was used to screen for single nucleotide polymorphisms in the spike gene (leading to amino acid changes N501Y and del69/70) associated with variants of concern such as B.1.1.7; both changes were detected (*6*).

Antibody Assessment and IFN-γ Release of SARS-CoV-2–Specific T Cells

For detection of SARS-CoV-2–specific antibodies to the spike and nucleocapsid proteins, we used a microarray-based multiparameter immunoassay according to manufacturer's instructions (SeraSpot Anti-SARS-CoV-2 IgG, Seramun Diagnostica GmbH, https://www.seramun.com), as described elsewhere (*7*). We applied a commercially available IGRA for assessment of IFN-γ release of SARS-CoV-2–specific T cells according to manufacturer's instructions (Euroimmun, https://www.euroimmun.com), as described previously (*7*).

Virus Isolation

Virus isolation was performed by using Vero E6 cells (ATCC CRL-1586). Vero E6 cells were maintained in a 5% CO_2 atmosphere at 37°C and cultured in Dulbecco's Modified Eagle Medium (Sigma Aldrich, https://www.sigmaaldrich.com), supplemented with 10% fetal bovine serum, 1% non-essential amino acids 100x concentrate, and 1% sodium pyruvate 100 mM (ThermoFisher Scientific, https://www.thermofisher.com) and split twice a week. Vero E6 cells were seeded at a density of 175,000 cells per well in 24-well plates 1 day before isolation. Virus isolation experiments were performed under Biosafety Level 3 (BSL-3) conditions with enhanced respiratory personal protective equipment. For virus isolation, the medium was removed and cells were rinsed once with 1x phosphate buffered saline (ThermoFisher Scientific) and inoculated with 200 µL of swap sample. After 1 hour incubation at 37°C, 800 µL of isolation medium (supplemented with 2% FBS, 1% penicillin-streptomycin, and 1% amphotericin B) was added to each well. Cells were monitored for cytopathic effect (CPE) for the following 3 days. As soon as CPE was visible or at day 3 post inoculation, viral RNA was quantified from the

supernatant of the inoculated cells. To ensure that viruses with lower replication capacities were not missed, all cultures were cultivated for an additional 3 days. At 6 days after inoculation, all cultures were reexamined for CPE. No CPE was visible in any negatively tested culture. In addition, all supernatants were passaged once by inoculating fresh and confluent Vero E6 cells with 100 µL of cell culture supernatant (taken at 3 days postinoculation) from the respective samples and monitored as previously described. For isolation of viral RNA, 50 µL of supernatant was diluted in 300 µL of MagNA Pure 96 external lysis buffer (Roche). All samples were heat inactivated for 10 minutes at 70°C before export from the BSL-3. Isolation and purification of viral RNA was performed using the MagNA Pure 96 System (Roche) according to the manufacturer recommendations. Viral RNA was quantified by using rRT-PCR (E gene assay) as previously described in Corman et al. (*4*). Positive isolation success was determined when CPE was visible and viral RNA concentrations were above a threshold of 100,000 genome equivalents per µL.

High-Throughput Sequencing of SARS-CoV-2 Genomes

Sufficient sample material (SARS-CoV-2 RNA concentration >10^4 copies/mL) was available for sequencing for 14 patients. We applied a PCR amplicon-based sequencing approach by using random hexamers and the SuperScript III Reverse transcription kit (ThermoFisher Scientific) according to manufacturer's instructions after a PCR amplification using the primer sets (V3) published by the Artic Network (https://github.com/artic-network/artic-ncov2019) (*8*). We set up a 25 µL PCR master mix by using the Q5 High-Fidelity DNA Polymerase kit (New England Biolabs, https://www.neb.com) with 5 µL 5x Q5 Reaction Buffer, 13.15 µL RNase-free water, 0.5 µL 10 mM dNTPs, 3.6 µL of either 10 µM primer pool 1 or 2, 2.5 µL cDNA and 0.25 µL Q5 High-Fidelity DNA Polymerase. We performed PCR by using a thermocycling protocol with initial denaturation at 98°C for 30 sec, followed by 35 cycles of 98°C for 15 sec, 65°C for 5 min, followed by a final 2-min extension step at 72°C. PCR products were pooled and purified by using KAPA Pure Beads (Roche) according to manufacturer's instructions. We used up to 5 ng DNA of purified PCR amplicons and the KAPA Frag Kit, followed by HTS library preparation using the KAPA Hyper Prep Kit (Roche) according to manufacturer's instructions. Sequencing was done using the V3 chemistry (2x75bp) on the Illumina NextSeq platform (Illumina, https://www.illumina.com).

Statistics

Values are given as medians and interquartile range unless stated otherwise. GraphPad PRISM statistics version 27.0 (IBM Deutschland, https://www.ibm.com/de-de) was used for statistical analysis. Group differences were assessed in a univariate analysis by using Fisher exact test or nonparametric Mann Whitney U test. P values of <0.05 were considered statistically significant. All 95% CI for proportions were calculated by using the Wilson procedure with correction for continuity (*9*).

Bioinformatics

Reads were trimmed by using AdapterRemoval version 2.3.0 and aligned to the Wuhan-Hu-1 (GenBank Accession no. MN908947.2) reference sequence using bowtie2 (version 2.4.1). Consensus calling used ivar version 1.9 requiring a coverage of ≥3 reads per position, and a minimum frequency threshold of 0.6. Lineages were assigned using pangolin version 2.3.5 (https://github.com/hCoV-2019/pangolin). A phylogenetic tree was inferred from an alignment generated in MAFFT version 7.471, including all complete sequences from the outbreak as well as representative B.1.1.7 sequences from Berlin deposited in GISAID, using IQTree version 2.0.3 with a GTR substitution model and 10000 ultra-fast bootstrap replicates. Sequences are available on GISAID under accession numbers EPI_ISL_1635432 (H07), EPI_ISL_1635435 (H09), EPI_ISL_1635666 (H11), EPI_ISL_1635964 (H12), EPI_ISL_1636216 (H14), EPI_ISL_1636365 (H15), EPI_ISL_1636400 (H17), EPI_ISL_1636401 (H18), EPI_ISL_1636403 (H22), and EPI_ISL_2134632 (H25).

References

1. Kurth F, Roennefarth M, Thibeault C, Corman VM, Müller-Redetzky H, Mittermaier M, et al. Studying the pathophysiology of coronavirus disease 2019: a protocol for the Berlin prospective COVID-19 patient cohort (Pa-COVID-19). Infection. 2020;48:619–26. PubMed https://doi.org/10.1007/s15010-020-01464-x

2. Corman VM, Haage VC, Bleicker T, Schmidt ML, Mühlemann B, Zuchowski M, et al. Comparison of seven commercial SARS-CoV-2 rapid point-of-care antigen tests: a single-centre laboratory evaluation study. Lancet Microbe. 2021 Apr 7 [Epub ahead of print]s. PubMed https://doi.org/10.1016/S2666-5247(21)00056-2

3. Wölfel R, Corman VM, Guggemos W, Seilmaier M, Zange S, Müller MA, et al. Virological assessment of hospitalized patients with COVID-2019. Nature. 2020;581:465–9. PubMed https://doi.org/10.1038/s41586-020-2196-x

4. Corman VM, Landt O, Kaiser M, Molenkamp R, Meijer A, Chu DK, et al. Detection of 2019 novel coronavirus (2019-nCoV) by real-time RT-PCR. Euro Surveill. 2020;25:2000045. PubMed https://doi.org/10.2807/1560-7917.ES.2020.25.3.2000045

5. Matheeussen V, Corman VM, Donoso Mantke O, McCulloch E, Lammens C, Goossens H, et al.; RECOVER project and collaborating networks. International external quality assessment for SARS-CoV-2 molecular detection and survey on clinical laboratory preparedness during the COVID-19 pandemic, April/May 2020. Euro Surveill. 2020;25:2001223. PubMed https://doi.org/10.2807/1560-7917.ES.2020.25.27.2001223

6. Centers for Disease Control and Prevention. SARS-CoV-2 variant classifications and definitions. 2021 [cited 2021 May 8]. https://www.cdc.gov/coronavirus/2019-ncov/cases-updates/variant-surveillance/variant-info.html

7. Schwarz T, Tober-Lau P, Hillus D, Helbig ET, Lippert LJ, Thibeault C, et al. Delayed antibody and T-cell response to BNT162b2 vaccination in the elderly, Germany. Emerg Infect Dis. 2021 Jun XX [Epub ahead of print]. https://doi.org/10.3201/eid2708.211145

8. Muller N, Kunze M, Steitz F, Saad NJ, Mühlemann B, Beheim-Schwarzbach JI, et al. Severe acute respiratory syndrome coronavirus 2 outbreak related to a nightclub, Germany, 2020. Emerg Infect Dis. 2020;27:645–8. PubMed https://doi.org/10.3201/eid2702.204443

9. Newcombe RG. Two-sided confidence intervals for the single proportion: comparison of seven methods. Stat Med. 1998;17:857–72. PubMed https://doi.org/10.1002/(SICI)1097-0258(19980430)17:8<857::AID-SIM777>3.0.CO;2-E

10. Böhmer MM, Buchholz U, Corman VM, Hoch M, Katz K, Marosevic DV, et al. Investigation of a COVID-19 outbreak in Germany resulting from a single travel-associated primary case: a case series. Lancet Infect Dis. 2020;20:920–8. PubMed https://doi.org/10.1016/S1473-3099(20)30314-5

Appendix Table. Patient characteristics in outbreak of severe acute respiratory syndrome coronavirus 2 B.1.1.7 lineage after vaccination in a long-term care facility, Germany, February–March 2021*

Characteristics	All patients	Vaccinated patients	Unvaccinated patients	p value
No. patients	24 (100.00)	20 (83.33)	4 (16.67)	
Sex				
F	19 (79.17)	15 (75.00)	4 (100.00)	0.544
M	5 (20.83)	5 (25.00)	0	
Median age, y (range)	90 (75–105)	90.5 (75–105)	88.5 (84–94)	0.735
Underlying conditions				
Hypertension	17 (70.83)	15 (75.0)	2 (50.0)	0.552
Type 2 diabetes	7 (29.17)	6 (30.0)	1 (25.0)	1.000
COPD	5 (20.83)	4 (20.0)	1 (25.0)	1.000
Chronic kidney disease	10 (41.67)	7 (35.0)	3 (75.0)	0.272
Dementia	12 (50.00)	10 (50.0)	2 (50.0)	1.000
Other	20 (83.33)	16 (80.0)	4 (100.0)	1.000
Infected	20 (83.33)	16 (80.0)	4 (100.0)	1.000
Outcome in infected patients				
Respiratory symptoms	9 (45.00)	5 (31.25)	4 (100.0)	0.026
Hospitalization	6 (30.00)	2 (12.5)	4 (100.0)	0.003
Oxygen in hospital	4 (20.00)	1 (6.25)	3 (75.0)	0.013
Oxygen after discharge	2 (10.00)	1 (6.25)	1 (25.0)	0.368
Death	2 (10.00)	2 (12.5)	0	1.000
Virologic examinations, median (IQR)				
Peak virus concentration, RNA copies/mL, log_{10}	6.62 (5.55–7.92)	6.45 (4.58–7.17)	8.15 (6.78–8.81)	0.100
Time to negative PCR or AgPOCT, d	10 (7–24)	7.5 (7.-17.25)	31 (21.50–34.50)	0.003

*Values are no. (%) except as indicated. AgPOCT, antigen point-of-care test; COPD, chronic obstructive pulmonary disease; IQR, interquartile range.

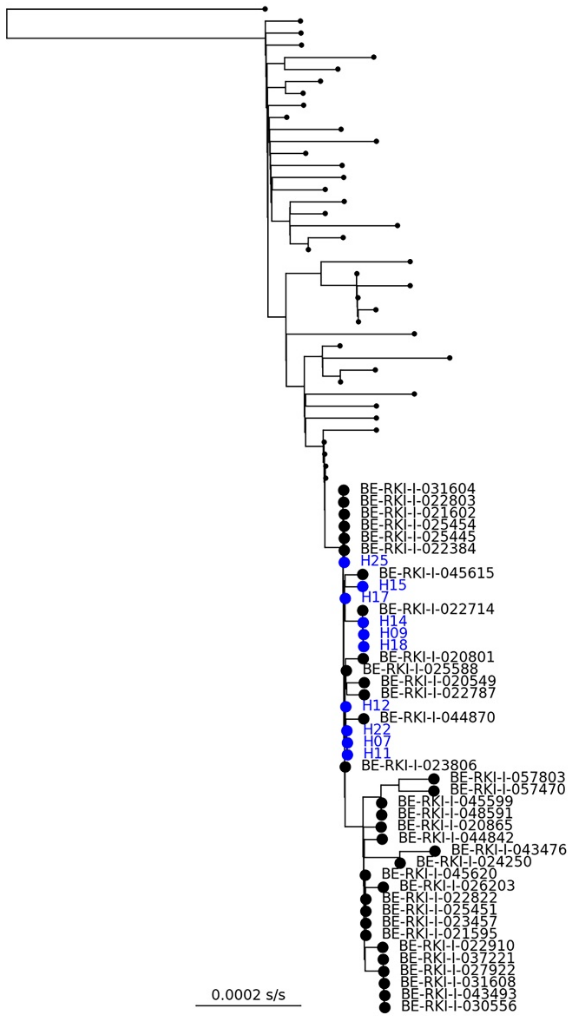

Appendix Figure 1. Maximum likelihood phylogenetic tree showing the sequences associated with outbreak of severe acute respiratory syndrome coronavirus 2 B.1.1.7 lineage in long-term care facility in relation to other B.1.1.7 sequences circulating in Berlin, January–February 2021. Complete genome sequences from the outbreak (n = 10) are in blue; additional sequences from Berlin are in black. For 4 samples, sequencing failed. Labels are given to sequences not associated with the outbreak if they fall into the same clade as the outbreak sequences. The x-axis shows substitutions per site. Asterisks indicate nodes with bootstrap support >70 and nodes with bootstrap support ≤30 are shown as polytomies. The tree is rooted with an early sequence from Wuhan (GISAID accession no. EPI_ISL_402125). The complete sequences from 6 patients are identical (H07, H11, H12, H17, H22, and H25). One patient had a mutation not present in any other sequence (H15) and 4 patients had a variable genome sequence position with a nucleotide that was present either as a major (H09, H14, H18) or a minor (H17) variant. The new mutation in H15 is likely the result of sporadic mutation (*10*); the variant present (sometimes as a minority) in 4 patients may be indicative of direct transmission links within the unit.

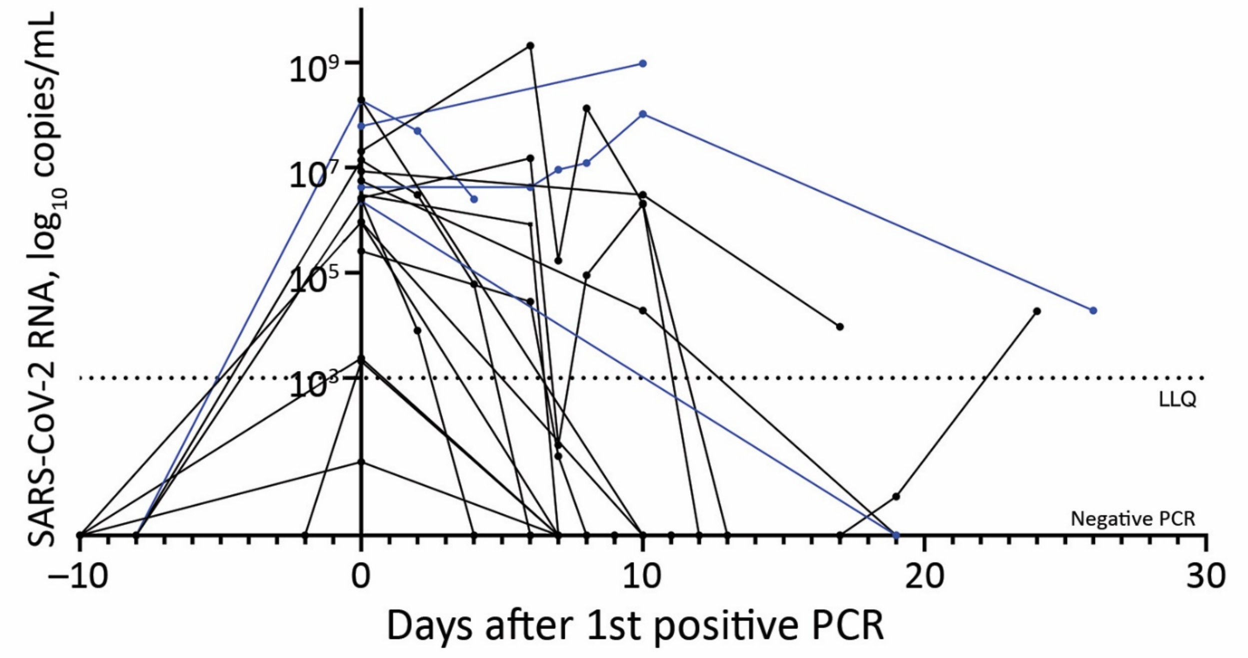

Appendix Figure 2. SARS-CoV-2 RNA viral concentration/mL of diluted swab specimens from vaccinated (n = 20) and unvaccinated (n = 4) residents of long-term care facility over 30 days, Germany, February–March 2021. Assessment of SARS-CoV-2 RNA concentration was done by applying external or internal calibration curves and quantified SARS-CoV-2 RNA and by using serial diluted specific in-vitro transcribed RNA. Unvaccinated residents are shown in blue. LLQ, lower limit of quantification; SARS-CoV-2, severe acute respiratory syndrome coronavirus 2.

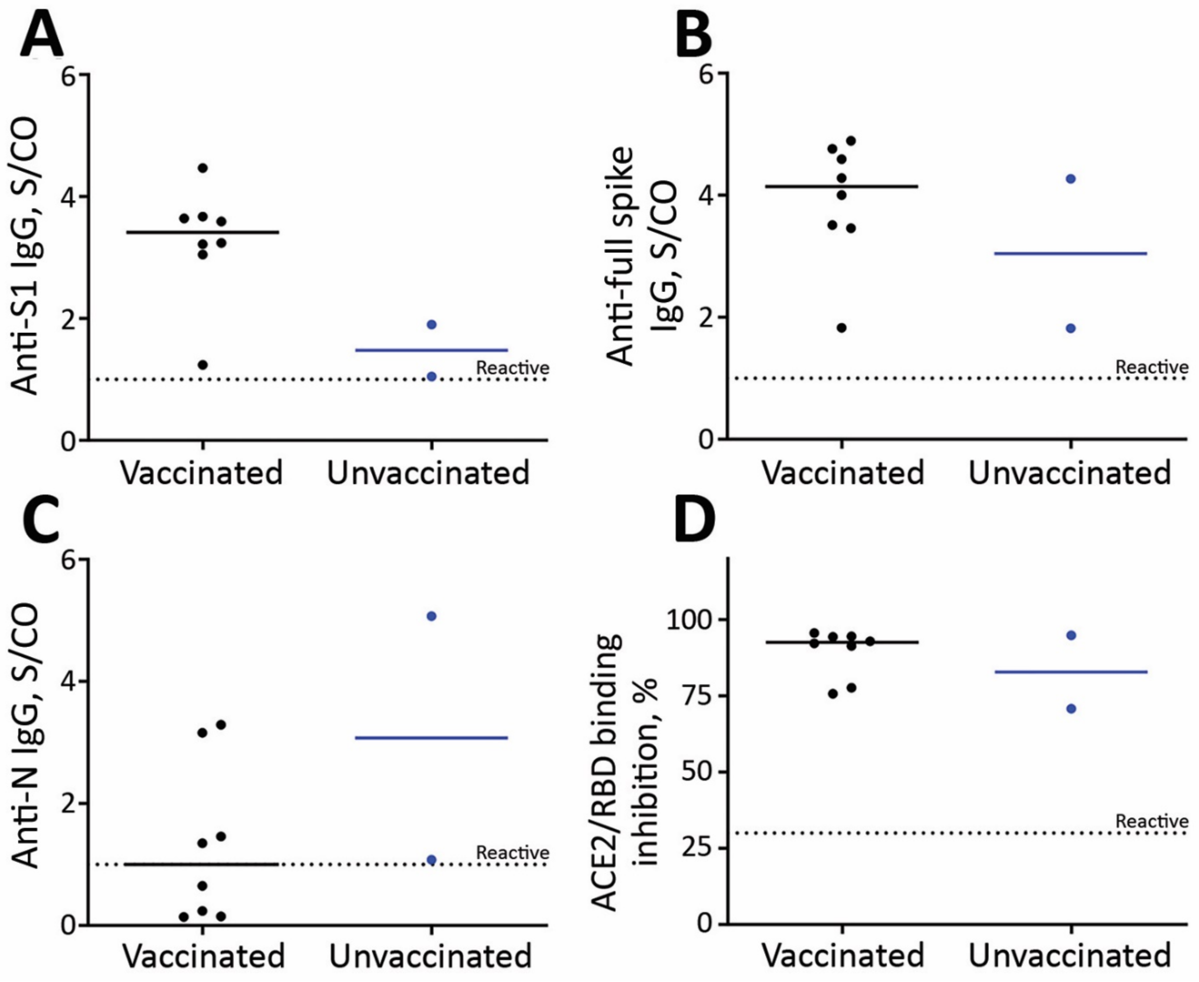

Appendix Figure 3. Anti-SARS-CoV-2 S1, full spike, and nucleocapsid IgG antibody response and surrogate virus neutralization test after vaccination in study of outbreak of SARS-CoV-2 B.1.1.7 lineage after vaccination in long-term care facility, Germany, February–March 2021. A) Anti-SARS-CoV-2 S1, B) full spike, and C) nucleocapsid-specific IgG antibodies were measured in 10/20 (50.00%) vaccinated and 2/4 (50.00%) unvaccinated residents 5 weeks after initial testing. D) Neutralizing capacity of antibodies was measured using the ELISA-based surrogate virus neutralization test cPASS (medac GmbH, https://international.medac.de). Unvaccinated patients are shown in blue. ACE2, angiotensin-converting enzyme 2; RBD, receptor-binding domain; SARS-CoV-2, severe acute respiratory syndrome coronavirus 2; S/CO, signal-to-cutoff ratio.
